# Simple Chemoinformatics Criterion Using Electron Donor-Acceptor Molecular Characteristics for Selection of Antibiotics Against Multi-Drug-Resistant Bacteria

**DOI:** 10.15190/d.2016.11

**Published:** 2016-10-01

**Authors:** Veljko Veljkovic, Sanja Glisic, Vladimir Perovic, Slobodan Paessler, Nevena Veljkovic, Garth L. Nicolson

**Affiliations:** Biomed Protection, Galveston, TX, USA; Center for Multidisciplinary Research, University of Belgrade, Institute of Nuclear Sciences VINCA, P.O. Box 522, 11001 Belgrade, Serbia; Department of Pathology, Galveston National Laboratory, University of Texas Medical Branch, 301 University Boulevard, Galveston, TX, USA; Department of Molecular Pathology, The Institute for Molecular Medicine, Huntington Beach, CA, USA

**Keywords:** Bacteria, multi-drug resistance, antibiotics, molecular descriptors

## Abstract

Recent outbreaks of NDM-1-positive Entero-bacteriaceae in Great Britain and India and the highly pathogenic Escherichia coli strain EHEC O104:H4 in Germany and some other E.U. countries point out an urgent need for the ability to decide on appropriate antibiotics to treat multi-drug-resistant (MDR) bacteria. Here we propose a simple criterion for choosing antibiotics based on characteristics of electron donor and acceptor properties. Using molecular descriptors, such as electron-ion interaction potential (EIIP) and average quasi-valence number (AQVN), which can describe potential long-range interactions between therapeutic molecules and their targets, we have been able to suggest appropriate antibiotics for treatment of MDR bacterial infections. To demonstrate the prospective usefulness of these molecular descriptors we have used this informatics system to propose that pleuromutilins and nitrofurans could be effective against of NDM-1-positive Enterobacteriacea and that aminoglycosides, macrolides and pluromutilins (and possibly nitrofurans) could be suitable for treatment of the highly pathogenic Escherichia coli strain EHEC O104:H4. Similarly, because of their specific electronic properties, we can also suggest antibiotics that could be potentially effective against other MDR bacteria. The proposed antibiotics should be further evaluated for their treatment potentials.

## 1. Introduction

The worldwide morbidity and mortality rates attributed to multi-drug-resistant (MDR) pathogens have been increasing rapidly. According to the WHO, between 2011 and 2015 more than 2 million people have been diagnosed with MDR bacterial infections^1^. In the case of MDR-*Mycobacterium tuberculosis* (MDR-TB), more than 25,000 diagnoses are confirmed each year^1^. In addition, the emergence in 2010 of new MDR gram-negative Enterobacteriaceae with resistance to carbapenem, conferred by New Delhi metallo-β-lactamase 1 (NDM-1), was heralded as a potential major global health problem due to resistance to most if not all antibiotics^2^. Recent outbreaks in Germany and other E.U. countries of Enterohaemorrhagic *E. coli *(EHEC O104:H4) bacteria that are resistant to a broad spectrum of antibiotics, as well as cases of haemolytic uraemic syndrome (HUS), have emphasized the urgent need for effective antibiotics for treatment of severe infections caused by MDR bacteria^3^.

Previously, the molecular descriptors that determine long-range interactions between therapeutic molecules and their molecular targets, such as the electron-ion interaction potential (EIIP) and average quasi-valence number (AQVN)^4,5^, were used for the discovery of potential new molecules with anti-HIV activities^6-8^. Here, we have used these same molecular descriptors to analyze antibiotics for their potential use against MDR bacteria, such as NDM-1-positive *Enterobacteriaceae* and *E. coli *strain**EHEC O104:H4. Based on results of this analysis, we have proposed a simple and general chemoinformatics criterion for selection of antibiotics that could be potentially effective for treatment of MDR bacteria.

## 2. Descriptors for Molecular Interactions

The physical concept of using the EIIP and AQVN to describe molecular interactions was previously described in detail elsewhere^4,8^. Here we will briefly present how calculation of the molecular descriptors (EIIP and AQVN) can be used for organic molecules, such as antibiotics, by the use of a simple equation derived from the “general model of pseudopotential”^9-11^.

The molecular descriptor EIIP (W) is defined by the equation^9^,

$$
W = 0.25 \frac{Z^ \star sin(1.04  \pi  Z^ \star ) }{2 \pi } 
$$ (1)

and Z* is the average quasi-valence number (AQVN) determined by

$$
Z^ \star =  \frac{1}{N}   \sum_{i=1}^{m}{n_i Z_i} 
$$ (2)

where Z*i *is the valence number of the *i*-th atomic component, *n_i_*is the number of atoms of the *i*-th component, *m *is the number of atomic components in the molecule, and N is the total number of atoms.

The EIIP values can be calculated according to equations (1) and (2) in Rydbergs (Ry).

## 3. Results Using Molecular Descriptors EIIP and AQVN

A strong connection between EIIP and AQVN calculated for organic molecules and their biological activities (mutagenicity, carcinogenicity, toxicity as well as antibiotic, cytostatic and anti-HIV activities) has been demonstrated (for reviews, see references^4,9^). It was shown that these biological activities, which are usually conferred by the ability of a molecule or its metabolites to interact covalently or non-covalently with various cellular or extra-cellular targets, are influenced by molecular electronic properties, such as EIIP and AQVN, that determine long-range intermolecular interactions^4^.

Of important note is that the molecular descriptors that we have described above do not depend on knowing precise molecular structures (see Equations [1] and [2]). This suggests that long-distance recognition and targeting between interacting molecules are structurally invariant. Thus, the EIIP and AQVN are parameters, among over 3,300 molecular descriptors, that are currently being used for characterization of organic molecules^5^. These parameters can describe potential long-range molecular interaction properties of molecules.

Here we have used the EIIP and AQVN parameters for analysis of different antibiotic classes and individual antibiotics for MDR *Enterobacteriaceae* and *E. coli *strain**EHEC O104:H4. In order to determine the range of molecular descriptors for each of the antibiotic classes that we analyzed, we have presented the values of EIIP and AQVN calculated for 230 penams, cephems, carbapenems and penems, monobactams, β-lactamase inhibitors, quinolones, aminoglycosides, ansamycins, tetracyclines, macrolides, pleuromutilins, sulfonamides, rifamycins, lincosamides, glycopeptides, nitromidizoles, oxazolidinones, lipopeptides, streptogramins and nitrofurans, among other possible antibiotics (Supplementary Table 1). The ranges of EIIP and AQVN descriptors, which encompass >85% of the antibiotics of each analyzed class, are shown in**Table 1**. In order to locate the positions of the analyzed antibiotics in the chemical spaces represented by EIIP and AQVN, we have compared the calculated molecular descriptors with the same molecular descriptors calculated for 45,010,644 compounds from the PubChem database (http://www.ncbi.nlm.nih.gov/pccompound).

**Figure 1:**
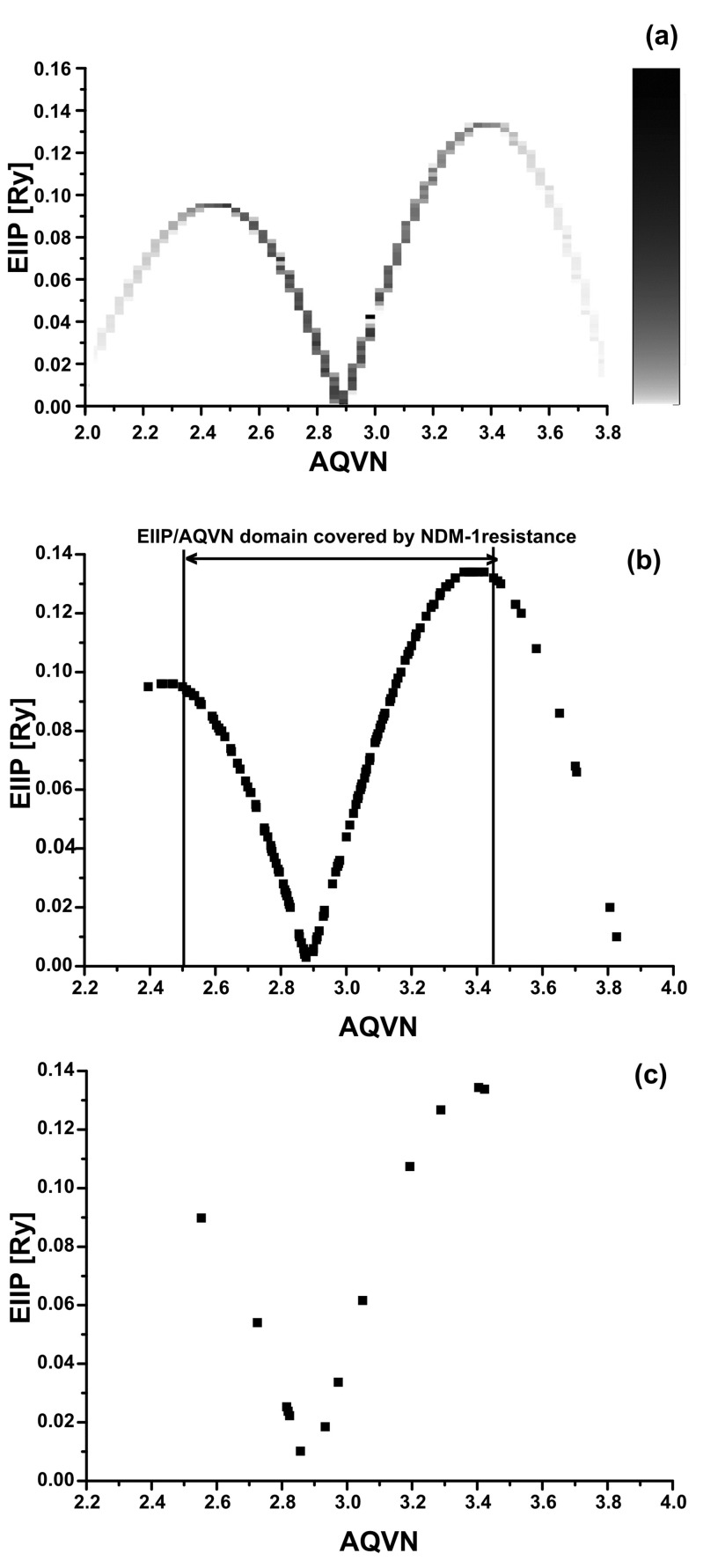
Distribution of EIIP and AQVN values calculated for (a) 44,987,581 compounds from PubChem database, (b) antibiotics presented in Supplementary Table 1, and (c) antibiotics for which NDM-1-positive Entero-bacteriaceae isolated in Great Britain and India are resistant^2^.

As shown in**Figure 1**, 92.5% of the compounds from the PubChem database are homogenously distributed within EIIP and AQVN intervals 0.00 – 0.11 Ry and 2.4 – 3.3, respectively. This domain of the EIIP/AQVN space, encompassing the majority of known chemical compounds, will be further referred to as the “basic EIIP/AQVN chemical space” (BCS). In**Figure 1** (b) the distribution of EIIP and AQVN values of the antibiotics from Supplementary Table 1 are shown. Note that the overwhelming majority of analyzed antibiotics (94.3%) are located within the BCS.

**Table 1:** Ranges of AQVN and EIIP that are characteristic for particular antibiotic classes

Antibiotic class	AQVN	EIIP [Ry]
Penicillins	2.975 – 3.180	0.035 – 0.124
Cephalosporins	3.071 – 3.473	0.070 – 0.130
Carbapenems & Penems	2.973 – 3.059	0.022 – 0.066
Monobactams	3.166 – 3.581	0.100 – 0.134
Quinolines	2.760 – 3.060	0.003 – 0.065
Aminoglycosides	2.552 – 2.820	0.024 – 0.084
Tetracyclines	2.933 – 3.111	0.018 – 0.084
Macrolides	2.467 – 2.630	0.077 – 0.096
Pleuromutilins	2.395 – 2.473	0.095 – 0.096
Nitrofurans	3.652 – 3.826	0.010 – 0.086

## 4. Discussion

Previously we demonstrated that the specific AQVN/EIIP domains combined with the structural properties of molecules can be used as a possible filter for the virtual screening of molecular libraries for new active drug candidates^4,6-8^. Accordingly, the AQVN/EIIP intervals presented in**Table 1**, representing the chemoinformatic “fingerprints” of various antibiotic classes, can be used for the virtual screening of molecular databases for new candidate antibiotics. Once new possible antibiotic candidates are identified, traditional methods can be used to confirm their usefulness. Thus, expensive testing on thousands of potential new antibiotics could be reduced to manageable numbers.

**Figure 2:**
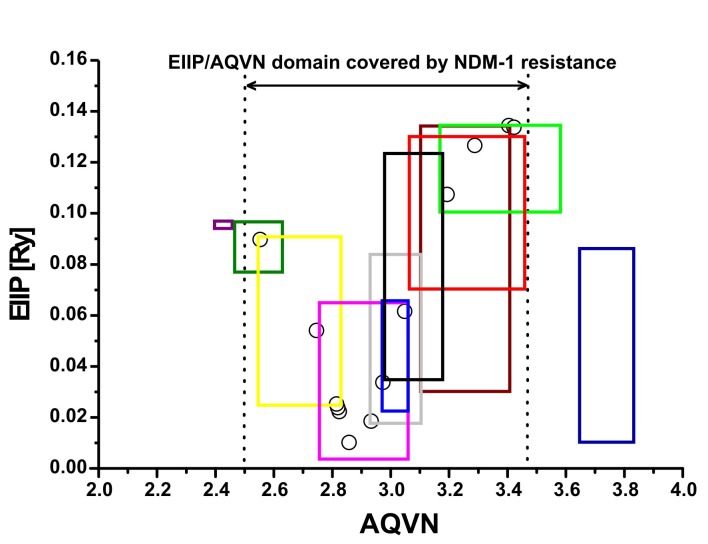
The EIIP/AQVN domains of major antibiotic classes. Each of presented domains encompasses >85% compounds belonging to the particular antibiotic class (black, penicillins; green, cephalosporins; gray, carbapenems and penems; magenta, quinolines; yellow, aminoglycosides; red, tetracyclines; olive, macrolides; royal, nitrofurans; purple, pleuromutilins; wine, sulfonamides. Open circles identify antibiotics for which NDM-1-positive Enterobacteriaceae isolated in Great Britain and North (Chennai) and South (Haryana) India are resistant.

The approach described here can also be used to identify potential new antibiotics that can overcome the problem of antibiotic resistance. For example, the EIIP and AQVN values can be calculated for antibiotics useful for treatment of resistant NDM-1-positive *Enterobacteriaceae* isolated in Great Britain and in North (Chennai) and South (Haryana) India (**Table 2**). In this example, the ‘resistant’ antibiotics are distributed within EIIP and AQVN ranges of 0.00 – 0.13 Ry and 2.55 – 3.42, respectively. As shown in**Figure 2**, these antibiotics cover the EIIP/AQVN area or domain of nearly all analyzed antibiotic classes, with the exception of pleuromutilins and nitrofurans. Therefore, the results presented in **Figure 1**(b, c) and**Figure 2**suggest that pleuromutilins and nitrofurans could be potentially useful antibiotics for overcoming the problem of antibiotic-resistance in NDM-1-positive Enterobacteriacea.

**Table 2:** Antibiotic susceptibilities for NDM-1-positive Enterobacteriaceae isolated in the UK and North and South India^2^

Antibiotic	Chemical Formula	AQVN	EIIP [Ry]	Susceptibility
Imipenem	C_12_H_17_N_3_O_4_S	2.973	0.0337	0%
Meropenem	C_17_H_25_N_3_O_5_S	2.8235	0.0223	3%
Piperacillin	C_23_H_27_N_5_O_7_S	3.0476	0.0616	0%
Cefotaxime	C_16_H_17_N_5_O_7_S_2_	3.4043	0.1344	0%
Ceftazidime	C_22_H_22_N_6_O_7_S_2_	3.2881	0.1267	0%
Cefpirome	C_22_H_22_N_6_O_5_S_2_	3.193	0.1074	0%
Aztreonam	C_13_H_17_N_5_O_8_S_2_	3.4222	0.1338	8%
Ciprofloxacin	C_17_H_18_FN_3_O_3_	2.8571	0.0102	8%
Gentamicin	C_21_H_43_N_5_O_7_	2.5526	0.0898	3%
Tobramycin	C_18_H_37_N_5_O_9_	2.7246	0.0541	0%
Amikacin	C_22_H_43_N_5_O_13_	2.8193	0.0238	0%
Minocycline	C_23_H_27_N_3_O_7_	2.9333	0.0185	0%
Tigecycline	C_29_H_39_N_5_O_8_	2.8148	0.0253	56%-67%

We can also compare the AQVN and EIIP descriptors calculated for 25 antibiotics that are in pre-clinical and clinical development for use against MDR bacteria (**Table 3**)^12^.

**Table 3:** Antibiotics against multidrug-resistant bacteria in pre-clinical and clinical development^12^

Compound	Formula	AQVN	EIIP [Ry]
Ceftaroline	C_24_H_25_N_8_O_10_PS_4_	3.472	0.1298
Telavancin	C_80_H_106_Cl_2_N_11_O_27_P	2.863	0.0079
Oritavancin	C_86_H_97_Cl_3_N_10_O_26_	2.928	0.0164
Dalbavancin	C_88_H_100_Cl_2_N_10_O_28_	2.947	0.0239
Iclaprim	C_19_H_22_N_4_O_3_	2.833	0.0188
Ceftobiprole	C_20_H_22_N_8_O_6_S_2_	3.276	0.1248
Amadacycline	C_21_H_18_N_2_O_5_S	3.149	0.0952
Delafloxacin	C_18_H_12_ClF_3_N_4_O_4_	3.143	0.0934
Nemonoxacin	C_20_H_25_N_3_O_4_	2.769	0.0406
Radezolid	C_22_H_23_FN_6_O_3_	2.909	0.0092
PZ-601	C_18_H_21_N_3_O_4_S_2_	3	0.0439
NXL 103	C_50_H_63_N_9_O_10_	2.788	0.0345
Torezolid	C_17_H_15_FN_6_O_3_	3.143	0.0934
WCK-771	C_19_H_21_FN_2_O_4_	2.808	0.0275
Zabofloxacin	C_19_H_20_FN_5_O_4_	2.98	0.0362
CEM-101	C_43_H_60_FN_6_O_10_	2.692	0.0631
BC-3205	C_32_H_51_N_2_O_5_S	2.472	0.0959
RWJ-416457	C_18_H_20_FN_5_O_3_	2.894	0.0034
Platensimycin	C_24_H_27_NO_7_	2.881	0.0012
PMX-30063	C_35_H_46_F_2_N_8_O_8_S	2.82	0.0235
APN-1252	C_22_H_23_N_3_O_3_	2.824	0.0223
NXL 104	C_25_H_31_FN_2_O_4_S_2_	2.738	0.0501
ACHN-490	C_15_H_20_FN_5_O_4_	2.889	0.0016
FR 264205	C_25_H_29_F_2_Cl_3_N_6_O_2_	2.627	0.078
BAL-30072	C_46_H_63_N_6_O_15_PS	2.864	0.0078

Only two compounds from this list (ceftaroline and BC-3205) have AQVN and EIIP values outside of the plotted domain in**Figure 2** that encompasses NDM-1 resistance, suggesting that antibiotics listed in**Table 3**, which are potentially effective against NDM-positive bacteria, could be identified in advance of clinical testing. We are not suggesting that antibiotics that do not meet the criteria of AQVN and EIIP descriptors should not be tested. We are suggesting that antibiotics that meet these criteria should be considered a testing priority.

**Figure 3:**
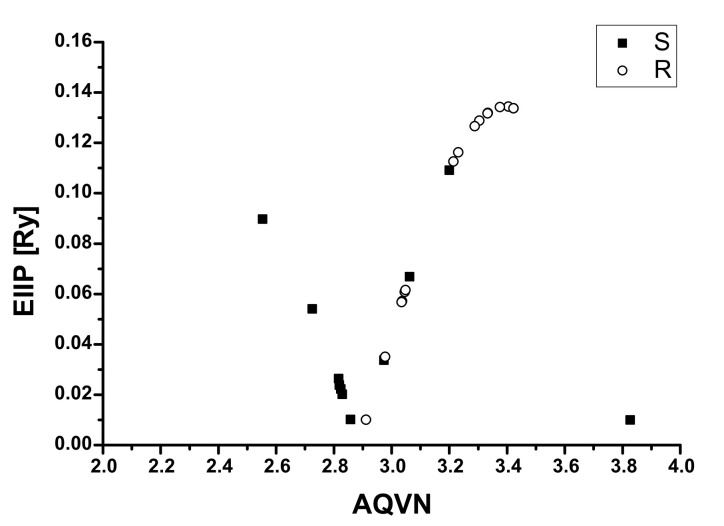
Distribution of AQVN and EIIP values of antibiotics tested against E. coli stain EHEC O104:H4^3^ (solid symbols, S=sensitive; open symbols, R=resistant).

Recently data on the sensitivities of antibiotics against the novel pathogenic *Escherichia coli* strain EHEC O104:H4, which caused a severe outbreak in Germany in May 2011, have been reported^3^. Using MDR strain EHEC O104:H4 bacteria the EIIP/AQVN descriptors have been calculated for various antibiotics that are listed in**Table 4**and shown as a domain plot in**Figure 3**. These data suggest that strain EHEC O104:H4 is sensitive to antibiotics with AQVN <2.9 and EIIP in the range of 0.01-0.10. Thus, the data suggest that MDR bacterial strain EHEC O104:H4 should be resistant to antibiotics outside of this AQVN and EIIP domain. Some exceptions to this rule, however, have been found: chloramphenicol, fosfomycin, imipenem and nitrofurantin. In addition, the data suggest that aminoglycosides, macrolides and pluromutilins could be highly effective against the pathogenic *Escherichia coli* strain EHEC O104:H4.

**Table 4:** Antibiotic resistance and molecular descriptors AQVN and EIIP of *E. coli *strain EHEC O104:H4

Antibiotic	AQVN	EIIP [Ry]	Resistance [x]*
Amoxicillin/Clavulanic acid	3.045/3.304	0.0608/0.1289	R
Cefuroxim	3.422	0.138	R
Piperacillin/Sulbactam	3.048/3.231	0.0616/0.1163	R
Tetracyclin	3.036	0.0572	R
Cefotaxim	3.404	0.1344	R
Ceftazidim	3.288	0.1267	R
Trimethoprim/Sulfamethoxazol	2.872/3.214	0.0048/0.1126	R
Amikacin	2.819	0.0238	S
Ciprofloxacin	2.875	0.0102	S
Tobramycin	2.725	0.0541	S
Chloramphenicol	3.062	0.0669	S
Fosfomycin	3.2	0.1092	S
Gentamicin	2.553	0.0898	S
Imipenem	2.973	0.0337	S
Kanamycin	2.816	0.0264	S
Meropenem	2.824	0.0223	S
Nalidixic acid	3.034	0.0568	R
Nitrofurantoin	3.826	0.01	S
Norfloxacin	2.829	0.0202	S
Piperacillin/Tazobactam	3.048/3.375	0.0616/0.1342	R
Streptomycin	2.911	0.0101	R
Ampicillin	2.977	0.0351	R
Cefoxitin	3.333	0.1319	R
Cefpodoxim	3.333	0.1319	R
Cefuroxim-Axetil	3.422	0.1338	R

Additionally, we found that the AQVN/EIIP domain of the NDM-1-containing antibiotic-resistant EHEC O104:H4 strain is broader than the corresponding domain found for EHEC O104:H4 strains that do not carry the NDM-1 gene. This suggests that acquisition of the NDM-1 gene by *Escherichia coli* EHEC O104:H4 could allow the selection of an appropriate antibiotic treatment against this highly pathogenic bacterial strain. For example, using the data presented in Table 5, there are two groups of antibiotics whose AQVN and EIIP descriptors are outside of the domain corresponding to the antibiotic resistance of the NDM-1-positive Enterobacteriacea and *Escherichia coli* strain EHEC O104:H4. These outlying antibiotics should be considered as therapeutic candidates for treatment of these MDR bacteria. It is of note that both groups of antibiotics are located outside the BCS and represent strong electron-donors (group A) and electron-acceptors (group B)^9^. Thus, therapies that include combinations of antibiotics from group A and B could have a greater potential for success, because simultaneous presentation of toxic compounds with significantly different electronic properties could represent an insurmountable problem for these bacteria. As a corollary, analysis of the AQVN and EIIP descriptors of antibiotics presented here suggest that: (i) pleuromutilins and nitrofurans could be effective against NDM-1-positive Enterobacteriacea; (ii) aminoglycosides, macrolides and pluromutilins (and possibly nitrofurans) could be suitable for treatment of the novel highly pathogenic *E. coli* strain EHEC O104:H4; and (iii) a combination of antibiotics from groups A and B could be considered as a therapeutic option for the treatment of multi-drug resistant bacterial infections (**Table 5**). These single and combination antibiotics need to be further evaluated for their treatment potentials against MDR bacterial strains.

**Table 5:** Potential antibiotics for treatment of MDR bacteria selected according to the AQVN/EIIP criterion.

Group I	Formula	AQVN	EIIP [Ry]
Cefpodoxime	C_15_H_17_N_5_O_6_S_2_	3.333	0.132
Tiamulin	C_28_H_47_NO_4_S	2.395	0.095
Retapamulin	C_30_H_47_NO_4_S	2.434	0.096
Valnemulin	C_31_H_52_N_2_O_5_S	2.44	0.096
Azithromycin	C_38_H_72_N_2_O_12_	2.468	0.096
BC-3205	C_32_H_51_N_2_O_5_S	2.472	0.096
Group II			
Ceftibuten	C_15_H_14_N_4_O_6_S_2_	3.463	0.131
Ceftaroline	C_24_H_25_N_8_O_10_PS_4_	3.472	0.13
Nifuroxazide	C_12_H_9_N_3_O_5_	3.517	0.123
Ceftriaxone	C_18_H_18_N_8_O_7_S_3_	3.518	0.123
Cefazolin	C_14_H_14_N_8_O_4_S_3_	3.535	0.12
Tigemonam	C_12_H_15_N_5_O_9_S_2_	3.581	0.108
Furazolidone	C_8_H_7_N_3_O_5_	3.652	0.086
Nitrofurazone	C_6_H_6_N_4_O_4_	3.7	0.068
Nifurtoinol	C_9_H_8_N_4_O_6_	3.704	0.066
Nifurzide	C_12_H_8_N_4_O_6_S	3.806	0.02
Nitrofurantoin	C_8_H_6_N_4_O_5_	3.826	0.01

## Bullet Points

**◊ There is an urgent need for new antibiotics to treat MDR bacterial infections**

**◊ We describe a simple set of chemoinformatic molecular descriptors based on characteristics of electron donor and acceptor properties to select new candidate antibiotics**

**◊ Using these molecular descriptors we have suggested appropriate antibiotics that could be suitable for treatment of the highly pathogenic, multi-drug-resistant bacteria**
