# Supplementary material for: Simple Chemoinformatics Criterion Using Electron Donor-Acceptor Molecular Characteristics for Selection of Antibiotics Against Multi-Drug-Resistant Bacteria
Source: Discoveries (Craiova). 2016 Oct 1;4(3):e64. doi: 10.15190/d.2016.11 (PMC7159820; doi:10.15190/d.2016.11)
Supplement: Supplementary file 1 [file discoveries-04-064-s001.pdf]

Discoveries Journals
publishing the future

DISCOVERIES 2016, Jul-Sep, 4(3): e64
DOI: 10.15190/d.2016.11

Supplementary Information

Simple Chemoinformatics Criterion Using Electron Donor-Acceptor Molecular Characteristics for Selection of Antibiotics Against Multi-Drug-Resistant Bacteria

Veljko Veljkovic[1,2], Sanja Glisic[2], Vladimir Perovic[2], Slobodan Paessler[3], Nevena Veljkovic[2], Garth L Nicolson[4]

[1] Biomed Protection, Galveston, TX, USA
[2] Center for Multidisciplinary Research, University of Belgrade, Institute of Nuclear Sciences VINCA, P.O. Box 522, 11001 Belgrade, Serbia
[3] Department of Pathology, Galveston National Laboratory, University of Texas Medical Branch, 301 University Boulevard, Galveston, TX, USA
[4]Department of Molecular Pathology, The Institute for Molecular Medicine, Huntington Beach, CA, USA

**Corresponding author*: Garth L Nicolson, PhD, MD (H), Department of Molecular Pathology, The Institute for Molecular Medicine, Huntington Beach, CA, 92647, USA; gnicolson@immed.org; Fax: 714-596-3791

Submitted: Sept. 23, 2016; *Revised:* Sept. 28, 2016; *Accepted:* Sept. 28, 2016; *Published:* Oct. 10, 2016;
Citation: Veljkovic V, Glisic S, Perovic V, Paessler S, Veljkovic N, Nicolson GL. Simple Chemoinformatics Criterion Using Electron Donor-Acceptor Molecular Characteristics for Selection of Antibiotics Against Multi-Drug-Resistant Bacteria. *Discoveries* 2016, Jul-Sep; 4(3): e64. DOI: 10.15190/d.2016.11

Supplementary Table 1. AQVN and EIIP values of selected antibiotics

Penicillins (Penams)

Antibiotic	Formula	AQVN	EIIP [Ry]
Narrow-spectrum			
β-lactamase sensitive			
Benzylpenicillin	$C_{16}H_{18}N_2O_4S$	2.976	0.035
Phenoxymethylpenicillin	$C_{16}H_{18}N_2O_5S$	3.048	0.062
Penicillinase-resistant			
Meticillin	$C_{17}H_{20}N_2O_6S$	3.043	0.060
Oxacillin	$C_{19}H_{19}N_3O_5S$	3.106	0.082
Nafcillin	$C_{21}H_{22}N_2O_5S$	2.980	0.036
Cloxacillin	$C_{19}H_{18}ClN_3O_5S$	3.106	0.082
Dicloxacillin	$C_{19}H_{17}Cl_2N_3O_5S$	3.106	0.082
Flucloxacillin	$C_{19}H_{17}ClFN_3O_5S$	3.106	0.082
β-lactamase-resistant			
Temocillin	$C_{16}H_{18}N_2O_7S_2$	3.244	0.119
Moderate-spectrum			
Amoxicillin	$C_{16}H_{19}N_3O_5S$	3.046	0.061
Ampicillin	$C_{16}H_{19}N_3O_4S$	2.977	0.035
Extended-spectrum			
Azlocillin	$C_{20}H_{23}N_5O_6S$	3.091	0.077

Carbenicillin	$C_{17}H_{18}N_2O_6S$	3.136	0.091
Ticarcillin	$C_{15}H_{16}N_2O_6S_2$	3.268	0.123
Mezlocillin	$C_{21}H_{25}N_5O_8S_2$	3.180	0.104
Piperacillin	$C_{23}H_{27}N_5O_7S$	3.048	0.062

Cephalosporins (Cephems)

First generation			
Cefalexin	$C_{16}H_{17}N_3O_4S$	3.073	0.071
Cefalotin	$C_{16}H_{16}N_2O_6S_2$	3.286	0.126
Cefazolin	$C_{14}H_{14}N_8O_4S_3$	3.535	0.120
Second generation			
Cefaclor	$C_{15}H_{14}ClN_3O_4S$	3.158	0.098
Cefuroxime	$C_{16}H_{16}N_4O_8S$	3.422	0.134
Cefamandole	$C_{18}H_{18}N_6O_5S_2$	3.306	0.129
Third generation			
Ceftriaxone	$C_{18}H_{18}N_8O_7S_3$	3.518	0.123
Cefotaxime	$C_{16}H_{17}N_5O_7S_2$	3.404	0.134
Cefpodoxime	$C_{15}H_{17}N_5O_6S_2$	3.333	0.132
Ceftazidime	$C_{22}H_{22}N_6O_7S_2$	3.288	0.127
Ceftibuten	$C_{15}H_{14}N_4O_6S_2$	3.463	0.131
Forth generation			
Cefepime	$C_{19}H_{24}N_6O_5S_2$	3.071	0.070
Cefpirome	$C_{22}H_{22}N_6O_5S_2$	3.193	0.107
Fifth generation			
Ceftaroline	$C_{24}H_{25}N_8O_{10}PS_4$	3.472	0.130

Carbapenems and Penems

Imipenem	$C_{12}H_{17}N_3O_4S$	2.973	0.034
Ertapenem	$C_{22}H_{25}N_3O_7S$	3.034	0.057
Meropenem	$C_{17}H_{25}N_3O_5S$	2.824	0.022
Faropenem	$C_{12}H_{15}NO_5S$	3.059	0.066
Doripenem	$C_{15}H_{24}N_4O_6S_2$	2.980	0.036

Monobactams

Aztreonam	$C_{13}H_{17}N_5O_8S_2$	3.422	0.134
Tigemonam	$C_{12}H_{15}N_5O_9S_2$	3.581	0.108
Nocardicin A	$C_{23}H_{24}N_4O_9$	3.167	0.100

β-lactamase inhibitors

Clavulanic acid	$C_8H_9NO_5$	3.304	0.129
Tazobactam	$C_{10}H_{12}N_4O_5S$	3.375	0.134
Clavulanic acid	$C_8H_9NO_5$	3.304	0.129

Quinolones

First generation			
Cinoxacin	$C_{12}H_{10}N_2O_5$	3.379	0.134
Flumequine	$C_{14}H_{12}FNO_3$	2.968	0.032
Nalidixic acid	$C_{12}H_{12}N_2O_3$	3.034	0.057
Oxolinic acid	$C_{13}H_{11}NO_5$	3.267	0.123
Piromidic acid	$C_{14}H_{16}N_4O_3$	2.973	0.034
Pipemidic acid	$C_{14}H_{17}N_5O_3$	2.974	0.034
Rosoxacin	$C_{17}H_{14}N_2O_3$	3.056	0.064
Second generation			
Ciprofloxacin	$C_{17}H_{18}FN_3O_3$	2.857	0.010
Enoxacin	$C_{15}H_{17}FN_4O_3$	2.900	0.006
Fleroxacin	$C_{17}H_{18}F_3N_3O_3$	2.773	0.039
Lomefloxacin	$C_{17}H_{19}F_2N_3O_3$	2.773	0.039
Nadifloxacin	$C_{19}H_{21}FN_2O_4$	2.808	0.028
Norfloxacin	$C_{16}H_{18}FN_3O_3$	2.829	0.020
Ofloxacin	$C_{18}H_{20}FN_3O_4$	2.870	0.006
Pefloxacin	$C_{17}H_{20}FN_3O_3$	2.773	0.039
Rufloxacin	$C_{17}H_{18}FN_3O_3S$	2.930	0.017
Third generation			
Balofloxacin	$C_{20}H_{24}FN_3O_4$	2.769	0.041
Gatifloxacin	$C_{19}H_{22}FN_3O_4$	2.816	0.025
Grepafloxacin	$C_{19}H_{22}FN_3O_3$	2.750	0.047
Levofloxacin	$C_{18}H_{20}FN_3O_4$	2.870	0.006
Moxifloxacin	$C_{21}H_{24}FN_3O_4$	2.792	0.033
Pazufloxacin	$C_{16}H_{15}FN_2O_4$	3.000	0.044
Sparfloxacin	$C_{19}H_{22}F_2N_4O_3$	2.760	0.044
Temafloxacin	$C_{21}H_{18}F_3N_3O_3$	2.875	0.004
Tosufloxacin	$C_{19}H_{15}F_3N_4O_3$	3.000	0.044
Fourth generation			
Clinafloxacin	$C_{17}H_{17}ClFN_3O_3$	2.857	0.010
Gemifloxacin	$C_{18}H_{20}FN_5O_4$	2.958	0.028
Sitafloxacin	$C_{19}H_{18}ClF_2N_3O_3$	2.826	0.021
Trovafloxacin	$C_{20}H_{15}F_3N_4O_3$	3.022	0.052
Prulifloxacin	$C_{21}H_{20}FN_3O_6S$	3.115	0.085

Ansamycins

Geldanamycin	$C_{29}H_{40}N_2O_9$	2.750	0.047
Herbimycin	$C_{30}H_{42}N_2O_9$	2.723	0.055

Aminoglycosides

Kanamycin	$C_{18}H_{36}N_4O_{11}$	2.812	0.026
Streptomycin	$C_{21}H_{39}N_7O_{12}$	2.911	0.010
Neomycin	$C_{23}H_{46}N_6O_{13}$	2.796	0.032
Paromomycin	$C_{23}H_{47}N_5O_{14}$	2.786	0.035
Amikacin	$C_{22}H_{43}N_5O_{13}$	2.819	0.024

Arbekacin	$C_{22}H_{44}N_6O_{10}$	2.707	0.059
Bekanamycin	$C_{18}H_{37}N_5O_{10}$	2.771	0.040
Dibekacin	$C_{18}H_{37}N_5O_8$	2.676	0.067
Tobramycin	$C_{18}H_{37}N_5O_9$	2.725	0.054
Spectinomycin	$C_{14}H_{24}N_2O_7$	2.808	0.028
Hygromycin B	$C_{20}H_{37}N_3O_{13}$	2.877	0.003
Gentamicin	$C_{21}H_{43}N_5O_7$	2.553	0.090
Netilmicin	$C_{21}H_{41}N_5O_7$	2.595	0.084
Sisomicin	$C_{19}H_{37}N_5O_7$	2.647	0.074
Isepamicin	$C_{22}H_{43}N_5O_{12}$	2.780	0.037
Verdamicin	$C_{20}H_{39}N_5O_7$	2.620	0.080
Astromicin	$C_{17}H_{35}N_5O_6$	2.603	0.082

Tetracyclines

Naturally-occurring			
Tetracycline	$C_{22}H_{24}N_2O_8$	3.036	0.057
Chlortetracycline	$C_{22}H_{23}ClN_2O_8$	3.036	0.057
Oxytetracycline	$C_{22}H_{24}N_2O_9$	3.088	0.076
Demeclocycline	$C_{21}H_{21}ClN_2O_8$	3.094	0.078
Semi-synthetic			
Doxycycline	$C_{22}H_{24}N_2O_8$	3.036	0.057
Lymecycline	$C_{22}H_{23}ClN_2O_8$	3.036	0.057
Meclocycline	$C_{22}H_{21}ClN_2O_8$	3.111	0.084
Metacycline	$C_{22}H_{22}N_2O_8$	3.111	0.084
Minocycline	$C_{23}H_{27}N_3O_7$	2.933	0.018
Rolitetracycline	$C_{27}H_{33}N_3O_8$	2.873	0.004

Glycylcycline

Tigecycline	$C_{29}H_{39}N_5O_8$	2.815	0.025

Streptogramins

Pristinamycin IA	$C_{45}H_{54}N_8O_{10}$	2.855	0.011
Pristinamycin IIA	$C_{28}H_{35}N_3O_7$	2.794	0.032
Quinupristin/dalfopristin	$C_{53}H_{67}N_9O_{10}S$	2.786	0.035
Virginiamycin S1	$C_{43}H_{49}N_7O_{10}$	2.899	0.005

Macrolides

Common macrolides			
Azithromycin	$C_{38}H_{72}N_2O_{12}$	2.468	0.096
Clarithromycin	$C_{38}H_{69}NO_{13}$	2.512	0.094
Dirithromycin	$C_{42}H_{78}N_2O_{14}$	2.500	0.095
Erythromycin	$C_{37}H_{67}NO_{13}$	2.525	0.093
Roxithromycin	$C_{41}H_{76}N_2O_{15}$	2.537	0.092

Telithromycin	$C_{43}H_{65}N_5O_{10}$	2.618	0.080
Developmental macrolides			
Carbomycin A	$C_{42}H_{67}NO_{16}$	2.667	0.069
Josamycin	$C_{42}H_{69}NO_{15}$	2.614	0.080
Kitasamycin	$C_{35}H_{59}NO_{13}$	2.611	0.081
Midecamycin	$C_{41}H_{67}NO_{15}$	2.629	0.078
Oleandomycin	$C_{35}H_{61}NO_{12}$	2.550	0.090
Spiramycin	$C_{43}H_{74}N_2O_{14}$	2.556	0.089
Troleandomycin	$C_{41}H_{67}NO_{15}$	2.629	0.078
Tylosin	$C_{46}H_{77}NO_{17}$	2.610	0.081
Ketolides			
Telithromycin	$C_{43}H_{65}N_5O_{10}$	2.618	0.080
Cethromycin	$C_{42}H_{59}N_3O_{10}$	2.649	0.073

Pleuromutilins

Tiamulin	$C_{28}H_{47}NO_4S$	2.395	0.095
Retapamulin	$C_{30}H_{47}NO_4S$	2.434	0.096
Valnemulin	$C_{31}H_{52}N_2O_5S$	2.440	0.096
BC-3205	$C_{32}H_{51}N_2O_5S$	2.472	0.096

Nitrofurans

Furazolidone	$C_8H_7N_3O_5$	3.652	0.086
Nitrofurantoin	$C_8H_6N_4O_5$	3.826	0.010
Nitrofurazone	$C_6H_6N_4O_4$	3.700	0.068
Nifurtoinol	$C_9H_8N_4O_6$	3.704	0.066
Nifuroxazide	$C_{12}H_9N_3O_5$	3.517	0.123
Nifurzide	$C_{12}H_8N_4O_6S$	3.806	0.020

Sulfonamides

Sulfadiazine	$C_{10}H_{10}N_4O_2S$	3.259	0.122
Sulfacetamide	$C_8H_{10}N_2O_3S$	3.167	0.100
Sulfamethizole	$C_9H_{10}N_4O_2S_2$	3.333	0.132
Sulfadimethoxine	$C_{12}H_{14}N_4O_4S$	3.200	0.109
Sulfamazone	$C_{23}H_{24}N_6O_7S_2$	3.226	0.115
Sulfamethoxazole	$C_{10}H_{11}N_3O_3S$	3.214	0.113
Prontosil	$C_{12}H_{13}N_5O_2S$	3.152	0.096
Sulfafurazole	$C_{11}H_{13}N_3O_3S$	3.097	0.079
Sulfadimidine	$C_{12}H_{14}N_4O_2S$	3.030	0.055
Sulfisomidine	$C_{12}H_{14}N_4O_2S$	3.030	0.055
Sulfametomidine	$C_{12}H_{14}N_4O_3S$	3.118	0.086
Sulfamoxole	$C_{11}H_{13}N_3O_3S$	3.097	0.079
Sulfaguanidine	$C_7H_{10}N_4O_2S$	3.167	0.100
Phthalylsulfathiazole	$C_{17}H_{13}N_3O_5S_2$	3.450	0.132
Succinylsulfathiazole	$C_{13}H_{13}N_3O_5S_2$	3.389	0.134
Aldesulfone	$C_{14}H_{16}N_2O_6S_3$	3.317	0.130

Trimethoprim	$C_{14}H_{18}N_4O_3$	2.872	0.005
Mafenide	$C_7H_{10}N_2O_2S$	3.000	0.044
Sulfametoxydiazine	$C_{11}H_{12}N_4O_3S$	3.226	0.115
Sulfapyridine	$C_{11}H_{11}N_3O_2S$	3.143	0.093
Sulfaperin	$C_{11}H_{12}N_4O_2S$	3.133	0.090
Sulfaphenazole	$C_{15}H_{14}N_4O_2S$	3.111	0.084
Sulfathiourea	$C_7H_9N_3O_2S_2$	3.304	0.129
Sulfaquinoxaline	$C_{14}H_{12}N_4O_2S$	3.212	0.112
Sulfadicramide	$C_{11}H_{14}N_2O_3S$	2.968	0.032
Sulfathiazole	$C_9H_9N_3O_2S_2$	3.360	0.134
Sulfasalazine	$C_{18}H_{14}N_4O_5S$	3.381	0.134

Rifamycins

Rifampicin	$C_{43}H_{58}N_4O_{12}$	2.752	0.046
Rifabutin	$C_{46}H_{62}N_4O_{11}$	2.699	0.061
Rifapentine	$C_{47}H_{64}N_4O_{12}$	2.709	0.059

Lincosamides

Clindamycin	$C_{18}H_{33}ClN_2O_5S$	2.533	0.092
Lincomycin	$C_{18}H_{34}N_2O_6S$	2.590	0.085

Cycloserine

Cycloserine	$C_3H_6N_2O_2$	3.077	0.072

Glycopeptides

Teicoplanins	$C_{77}H_{77}Cl_2N_9O_{13}$ -R<br>R: $C_{10}H_{17}O$, $C_{10}H_{19}O$, $C_{11}H_{21}O$	2.75	0.046
Vancomycin	$C_{66}H_{75}Cl_2N_9O_{24}$	3.011	0.048
Telavancin	$C_{80}H_{106}Cl_2N_{11}O_{27}P$	2.863	0.008

Nitromidiazoles

Metronidazole	$C_6H_9N_3O_3$	3.143	0.093
Tinidazole	$C_8H_{13}N_3O_4S$	3.103	0.081
Ornidazole	$C_7H_{10}ClN_3O_3$	3.000	0.044
Nimorazole	$C_9H_{14}N_4O_3$	2.933	0.019
Secnidazole	$C_7H_{11}N_3O_3$	3.000	0.044
Azanidazole	$C_{10}H_{10}N_6O_2$	3.286	0.126
Propenidazole	$C_{11}H_{13}N_3O_5$	3.188	0.106

Chloramphenicol

Chloramphenicol	$C_{11}H_{12}Cl_2N_2O_5$	3.062	0.067

Trimethoprim

Trimethoprim	$C_{14}H_{18}N_4O_3$	2.872	0.005

Mupirocin

Mupirocin	$C_{26}H_{44}O_9$	2.557	0.089

Oxazolidinones

Linezolid	$C_{16}H_{20}FN_3O_4$	2.818	0.024
Torezolid	$C_{17}H_{15}FN_6O_3$	3.143	0.093
Eperezolid	$C_{18}H_{23}FN_4O_5$	2.863	0.008
Posizolid	$C_{21}H_{21}F_2N_3O_7$	3.037	0.058
Radezolid	$C_{22}H_{23}FN_6O_3$	2.909	0.009

Lipopeptides

Surfactin	$C_{53}H_{93}N_7O_{13}$	2.518	0.093
Daptomycin	$C_{72}H_{101}N_{17}O_{26}$	2.917	0.012
Echinocandin B	$C_{52}H_{81}N_7O_{16}$	2.692	0.063
